# Interactions between Gut Microbiota, Host Circadian Rhythms, and Metabolic Diseases

**DOI:** 10.1016/j.advnut.2025.100416

**Published:** 2025-03-24

**Authors:** Mingliang Zhang, Caiyuan Zhou, Xinguo Li, Hui Li, Qi Han, Zhong Chen, Wenjie Tang, Jie Yin

**Affiliations:** 1 College of Animal Science and Technology, Hunan Agriculture University, Changsha, China; 2 Xi'an Tiankang Feed, Co, Ltd, Xian, China; 3 Hunan Institute of Animal and Veterinary Science, Changsha, China; 4 Xiangxi Vocational and Technical College for Nationalities, Jishou, China; 5 Animal Breeding and Genetics Key Laboratory of Sichuan Province, Sichuan Animal Science Academy, Chengdu, China; 6 Livestock and Poultry Biological Products Key Laboratory of Sichuan Province, Sichuan Animtche Group, Co Ltd, Chengdu, China

**Keywords:** circadian rhythms, gut microbiota, microbial metabolites, metabolic homeostasis, interaction

## Abstract

The circadian rhythm arises endogenously from genetically encoded molecular clocks, wherein the components collaborate to induce cyclic fluctuations, occurring approximately every 24 h. The rhythms synchronize biological processes with regular and predictable environmental patterns to guarantee the host metabolism and energy homeostasis function and well-being. Disruptions to circadian rhythms are widely associated with metabolic disorders. Notably, microbial rhythms are influenced by both the host’s intrinsic circadian clock and external rhythmic factors (i.e., light–dark cycle, diet patterns, and diet composition), which affect the structure of microbial communities and metabolic functions. Moreover, microbiota and the metabolites also reciprocally influence host rhythms, potentially impacting host metabolic function. This review aimed to explore the bidirectional interactions between the circadian clock, factors influencing host–microbial circadian rhythms, and the effects on lipid metabolism and energy homeostasis.

**Statement of significance**

This review explores the factors influencing both host and microbial rhythms, highlighting the interactions between gut microbiota, the metabolites, and host circadian rhythms. Additionally, it emphasizes the impact of disruptions in microbial and host rhythms on the development of metabolic diseases.

## Introduction

Most organisms have developed the circadian clock system to adapt to daily environmental cycles, driving periodic behavioral and physiologic oscillations in anticipation of geophysical time changes [1]. Circadian rhythms (exhibit a periodicity of ∼24 h) are vital to almost all forms of life due to coordinating crucial physiologic processes in most organisms [2,3]. The endogenous circadian clock synchronizes with external signals through zeitgebers, including light–dark cycles (the primary zeitgeber), food timing, type of food or drink, exercise, and temperature [2,4]. Thus, various factors, including jet lag, shift work, nighttime light, late-night eating, high-calorie food, and gene polymorphisms or behavior, do disrupt circadian rhythms [2,5,6].

Furthermore, the disturbance of the host–gut microbiota circadian system, caused by genetic, diet, or environmental factors, are linked to increased disease incidence and aggravated pathologic conditions [7,8]. Specifically, the gut microbiota comprises dynamic microbial communities, and the coevolution of the communities with the host has profoundly influenced host health [[9], [10], [11], [12], [13], [14]]. Recent research highlights the profound impact of trillions of gut microbes on host physiology, including digestion, absorption, metabolism, and energy balance, all of which are intricately linked to the host’s circadian clock [5]. It is not surprising that certain bacteria exhibit circadian rhythms, given the widespread significance of circadian rhythms across the microbiota [15,16].

Notably, the gut microbiota exhibits distinct circadian clock patterns, generating oscillations in critical metabolic mediators that integrate with the host’s circadian rhythm, contributing to metabolic homeostasis [16,17]. Disruptions in gut microbiota circadian rhythms harm host metabolism and energy balance, leading to metabolic syndrome [8,16]. Recent studies have highlighted the complex relationship between circadian rhythms and microbiota. This review aimed to explore the interactions between gut microbiota, host circadian rhythms, and lipid metabolism.

## Circadian Clocks

In mammals, the circadian system is a network of clocks led by the master clock in the suprachiasmatic nucleus (SCN) of the hypothalamus. This master clock is responsible for synchronizing or entraining peripheral clocks located in various tissues, such as the heart, liver, gastrointestinal tract, adipose tissue, and pancreas, thereby orchestrating behavioral and humoral rhythms throughout the organism [7,[18], [19], [20]]. The SCN, receiving environmental cues, centrally controls the host’s circadian rhythm and influences peripheral tissues via nerve signals and hormones [2,7]. Most tissues and peripheral organs in the mammalian body also express the equivalent clock genes and proteins to those present in the SCN [2].

**FIGURE 1:**
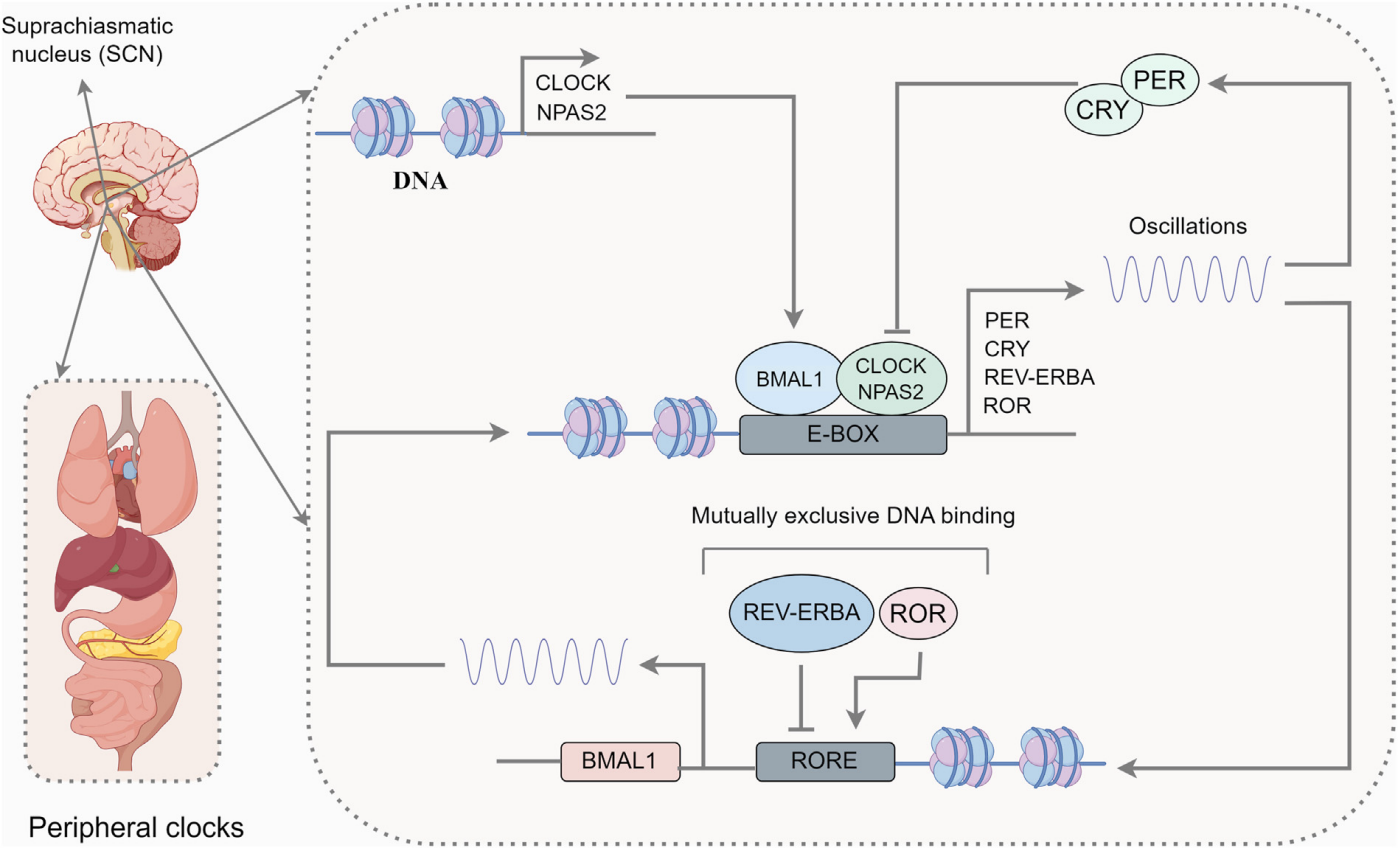
Typical molecular feedback loops of circadian rhythms. CLOCK-BMAL1 and NPAS2-BMAL1 heterodimers activate the transcription of *PER*, *CRY*, *REV-ERBA*, and *ROR* genes. PER and CRY inhibit CLOCK-BMAL1–dependent and NPAS2-BMAL1–dependent transcription. The REV-ERBA and ROR proteins are responsible for driving rhythmic BMAL1 transcription from ROR response elements (ROREs) in the promoter region. This figure was created using Figdraw.com.

Approximately, a 24-h cycle is generated at the molecular level by a cell-autonomous transcriptional autoregulatory feedback loop made up of clock genes [18,21] (Figure 1). The molecular clock involves 2 central components: the circadian locomotor output cycles kaput (CLOCK) and brain and muscle aryl hydrocarbon receptor nuclear translocator-like protein (BMAL) 1 (also known as ARNTL1) [1]. *Clock* and *BMAL1*, which encode activators, and Period (*Per*, comprising *Per1*, *Per2* and *Per3*) and cryptochrome (*Cry*, comprising *Cry1* and *Cry2*), which encode repressors, are the core clock genes [18].

In the primary feedback loop, *PER* and *CRY* genes are activated under the control of transcriptional activators: Clock, BMAL1, and neuronal PAS domain protein (NPAS) 2, which form CLOCK–BMAL1 and NPAS2–BMAL1 heterodimers, respectively [1]. These complexes activate transcription of the *PER* and *CRY* genes by binding to E-box (5'-CACGTG-3') elements in promoter regions [7]. Following the degradation of PER/CRY, CLOCK and BMAL1 begin transcriptional activation, whereupon a new circadian cycle can commence [7].

In a second molecular feedback loop, the CLOCK–BMAL1 complex drives the rhythmic expression of the genes encoding reverse erythroblastosis virus (REV-ERBs, REV-ERBα/β; also known as NR1D) proteins and retinoic acid–related orphan receptors (RORs; RORα/β/γ) families [1,7]. In contrast, REV-ERBα and RORα also contend for the ROR DNA-binding element located within the promoter regions of *Clock* and *Bmal1*, resulting in the repression or activation of transcription of *Clock* and *Bmal1*, respectively [1]. These complicated feedback loops generate rhythms lasting about a day and are called the circadian. Accordingly, understanding the biological processes improves our comprehension of circadian physiology and pathology.

## Endogenous Factors Influencing the Circadian Oscillation of Host Microbiota

**FIGURE 2:**
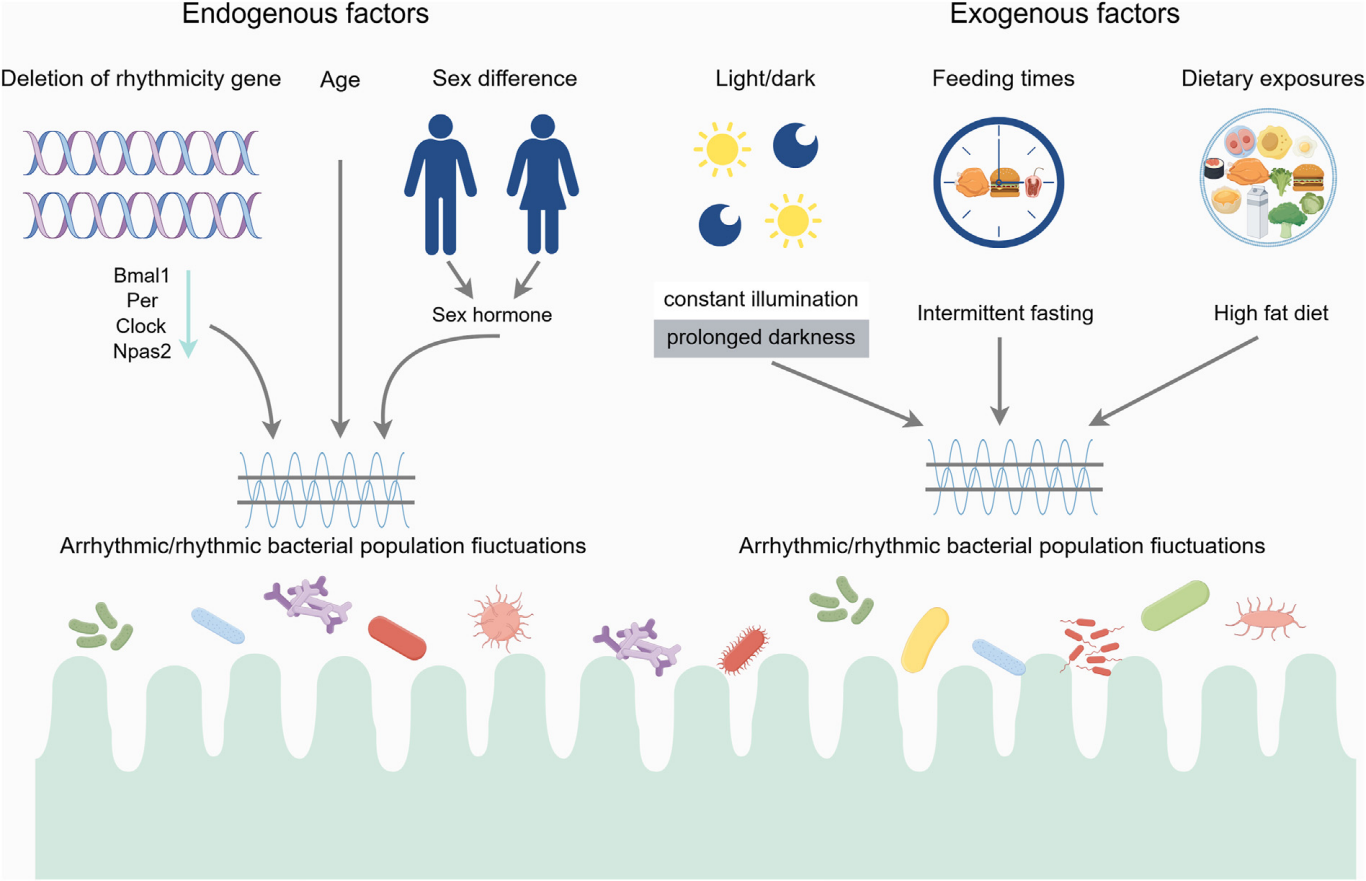
Endogenous and exogenous factors influence the circadian rhythms of the host–gut microbiota. The gut microbiota undergoes diurnal changes in composition and function driven by endogenous factors. Additionally, exogenous factors such as light/dark cycles, feeding times, and nutrient composition also impact the rhythmic oscillations of the host–gut microbiota. This figure was created using Figdraw.com.

To date, host circadian rhythms exhibit a significant influence on microbial oscillations, based on data from animal and human studies. Although gut microbiota is not exposed to light, alterations in host rhythms induce oscillations in the abundances and functions of gut microbiota [22] (Figure 2). However, the interactions between microbes and host factors that regulate circadian rhythm oscillations are complex and poorly understood.

### Deletion of rhythmic gene

The core molecular clock machinery can profoundly affect metabolic regulatory signals at the peripheral and central levels. Notably, deleting the host’s circadian clock gene disrupts the gut microbiota’s rhythm [23,24]. For example, *Bmal1* knockout (KO) mice exhibit altered diurnal fluctuations in the abundances of Bacteroidetes, Firmicutes, and Proteobacteria, along with increased abundances of *Rikenella* sp. [25]. Moreover, the rhythmicity of microbial metabolites, short-chain fatty acids (SCFAs), is eliminated in feces of *Bmal1*-deficient mice [26], indicating a role in microbial taxonomic abundance at multiple levels.

Interestingly, microbial α-diversity in *Per2* gene KO mice is notably higher than that in wild-type mice in a standard light–dark cycle [6]. Furthermore, the KO of the *Per2* gene leads to an increase Lachnospiraceae and Ruminococcaceae, although simultaneously decreasing concentrations of *Erysipelatoclostridium* and *Olsenella* species [6]. In addition, *Per1/2*-deficient mice display an almost complete absence of rhythmic fluctuations in the abundances of commensal bacteria, such as Bacteroidales [27]. Additionally, the diurnal rhythmicity in metagenomic pathways (i.e., vitamin and nucleotide metabolism and cell wall synthesis), observed in wild-type mice, disappear in *Per1/2*-deficient mice [27]. *Per3*-deficient mice also show alterations in the Shannon and Simpson indices, apart from a significant decrease in the abundance of *Dorea* species compared with the wild-type mice [24].

Notably, the average relative abundance of bacteria from the family Lachnospiraceae was elevated in Clock KO mice. This alteration contributed to an increased Firmicutes/Bacteroidetes ratio in *Clock* KO mice relative to wild-type mice [28]. Furthermore, conditional KO of *Npas2* in the liver alters the gut microbiota following restricted feeding [29]. These findings indicate that deletion of host circadian clock genes result in adverse disruptions to the gut microbiota [2,6,26].

### Sex affects the rhythms of gut microbiota

Current studies from both human and animal subjects have highlighted the significant role of sex as a contributing factor to the variation of gut microbiota [25,30]. Interestingly, changes in the circadian clocks of host organisms affect gut microbiota, and it has also been postulated that the effects differ between the sexes. For example, male and female mice in a controlled environment show significant differences in gut microbiota compositions [31]. To determine the role of sex hormones, testosterone was administrated and the results were characterized by the changes in gut microbiota caused by gonadectomy [31]. Moreover, the synthesis of steroids, including testosterone, is reported to be dependent on the circadian clock protein Bmal1 [32]. Therefore, hormones such as testosterone may be associated with different gut microbiota produced by the different sexes of the hosts.

Similarly, as well as the circadian system of the host, the sex of the host also influences the rhythmicity of microbial taxonomic abundances. The deletion of *Bmal1* leads to a disturbance of the host circadian clock, resulting in a sex-dependent alteration of the fecal microbial compositions [25]. Despite the microbial showed circadian rhythmicity is observed in both sexes, females show more significant oscillations than males [25]. Recent studies on male and female mice have demonstrated age-related alterations in the diurnal patterns of gut microbial compositions, with variations contingent upon the sex of the host [33]. In summary, alterations in host circadian rhythms significantly impact gut microbiota, potentially creating a feedback loop where gut microbes adjust their circadian activities [2].

## Exogenous Factors Affect the Circadian Oscillation of Host Microbiota

Exogenous factors, encompassing environmental cues and external stimuli, exert a profound influence on the circadian oscillation of gut microbes (Figure 2). These external factors not only modulate the timing but also determine the intensity of microbial activity, thereby profoundly shaping the dynamics of the microbial community within the host. Among the pivotal exogenous factors affecting circadian oscillation, light serves as a vital cue for numerous organisms, microbes included, in synchronizing the internal clocks. The mere presence or absence of light initiates specific molecular cascades within microbes, resulting in alterations in their metabolic processes and gene expression patterns. Furthermore, the abnormal diurnal fluctuations in the microbiota and dysbiosis are also driven by diet-related patterns [27]. Habitual diet, time-restricted feeding, late-night eating, rapid changes in fat and fiber composition of the diet, and consumptions of fiber and other indigestible food components have all been shown to affect the compositions, function, and rhythmicity oscillation of gut microbiota [2,5,[34], [35], [36], [37]].

### Light–dark cycle

A regular light–dark cycle is essential for circadian rhythms, nutrient metabolism, and gut microbiota homeostasis [6]. Predictively, prolonged darkness or constant illumination generally disrupts circadian rhythms [38,39]. The light cycles of the environment also entrain the circadian feeding behavior of animals, which produces rhythms in the exposure to food-borne bacteria [40]. Hence, light is the most important environmental factor affecting the circadian clock. For instance, mice fed a standard feed pellets diet lost the diurnal rhythmicity of gut microbes, as well as the oscillatory operational taxonomic units, when subjected to a phase-shift paradigm like jet lag [27]. Continuous darkness leads to the disappearance of certain families, such as Prevotellaceae [41]. Conversely, prolonged exposure to light induces alterations in Bacteroidetes and Firmicutes, which is correlated with weight gain and insulin resistance [39].

Interestingly, melatonin administration demonstrated a positive impact on ameliorating gut microbiota dysbiosis induced by continuous light exposure. Additionally, melatonin treatment not only reduces lipid content and improves insulin sensitivity but also decreases fat accumulation in the livers of mice on a high-fat diet exposed to constant light [39]. Similarly, the gut microbiota of mice exposed to regular light–dark cycles showed rhythmicity at the compositional and functional levels, but constant darkness resulted in the loss of rhythmic oscillations [38]. Notably, the abundance of *Clostridia* sp. in the small intestine is significantly elevated under conditions of constant darkness, indicating that the light–dark cycle plays a crucial role in regulating the concentration of *Clostridia* species [38]. However, *Ruminococcus torques* increased and *Lactobacillus johnsonii* decreased after 4 wk of constant 24-h light [42]. These findings underscore the critical role of the light–dark cycle in shaping the composition, functionality, and diurnal oscillations of the host microbiota.

### Diet patterns and diet composition

#### Intermittent fasting

The timing of food intake affects circadian rhythms, which regulate key physiologic processes essential for human health [43]. Currently, 3 types of intermittent fasting are extensively studied: whole-day fasting, alternate-day fasting, and time-restricted feeding [43,44]. However, due to its ease of integration into daily routines, time-restricted feeding has recently garnered significant attention [43]. Time-restricted feeding modulates nutritional cues that synchronize peripheral oscillations by limiting food intake to specific time intervals, such as an 8- to 10-h eating window, followed by a fasting period [45].

Time-restricted feeding has been shown to partially restore the cyclical fluctuations of gut microbes, thereby providing protection against obesity and metabolic disorders [46]. For instance, a time-restricted high-fat diet exhibited distinct circadian rhythms in the abundances of Bacteroidetes and Firmicutes [35]. Furthermore, mice subjected to a time-restricted high-fat diet exhibited significantly reduced weight gain, decreased hepatic steatosis, and lower hepatic triglyceride concentrations [35]. The mechanism may be due to time-restricted feeding altering gut microbes and molecular circadian rhythms associated with hepatic lipid metabolism. Likewise, time-restricted feeding may reduce obesity and metabolic risks by influencing circadian clock genes and the gut microbiome [47]. Time-restricted feeding modulates the circadian system by stimulating sirtuin 1 (sirtuin 1 regulates the circadian rhythms through the control of Bmal1 acetylation) [48] and increases gut microbial diversity, thereby ameliorating the serum lipid and liver profiles in healthy males [47]. Notably, time-restricted eating may lead to reduced calorie intake, and caloric restriction may also further impact circadian rhythms. For instance, caloric restriction reverses liver circadian genomic signatures of aging [49]. Interestingly, there are also differing viewpoints: time-restricted feeding is a dietary pattern based on circadian rhythm and do not require a significant reduction in calorie intake that was required for intermittent fasting and periodic fasting [50,51]. Hence, further study is needed to investigate the potential relationship between time-restricted feeding, caloric intake, and the regulation of the microbial circadian rhythm.

Furthermore, different feeding patterns influence clock–lipid–bile acid metabolic balance, particularly through gut microbiota, which connects circadian rhythms to bile acid metabolism [52]. For instance, daytime feeding, unlike ad libitum and dark-fed conditions, disrupts the liver’s rhythmic expression of *Per1*, *Cry1/2*, and *Rev-erb α*, and changes gut microbiota composition, increasing Firmicutes and decreasing Verrucomicrobia [52]. This suggests intermittent fasting has a regulatory effect.

#### Nutrient component

The nutrient component is a crucial signal for regulating circadian rhythms that target clock genes [53]. In addition, signals derived from the nutrition can also influence the rhythmicity of the expression of clock genes and their downstream targets, including metabolic genes [[54], [55], [56]]. High-fat diets have been shown to decrease gut microbiome α-diversity, reducing the number of microbial species with diel oscillation patterns [5,17,46,57]. For instance, high-fat diet increases the concentrations of Clostridiales, Peptostreptococcaceae, and certain *Lactobacillus* species in mice’s gut microbiota. Notably, exogenous melatonin administration enhances the composition and rhythmicity of gut microbes in mice subjected to a high-fat diet [34].

Furthermore, high-fat diets have the potential to affect the circadian rhythm of the gut microbiota by disrupting the rhythmic expression patterns of hepatic clock genes and adipose tissue clock genes, in addition to influencing downstream metabolic genes. In response to high-fat diets, the gut microbiota affected hepatic lipid metabolism, mainly by activating PPARγ signaling within the host’s circadian rhythm [58]. This suggests that a diet alone is insufficient in producing changes in host circadian rhythms unless it is coupled with a functional gut microbiota. Notably, disruptions in the circadian rhythm may also contribute to the development of obesity. Specifically, excessive caloric intake not only contributes to obesity but also realigns the circadian rhythms of peripheral organs, such as the liver and adipose tissue, further exacerbating the obesity [59]. Hence, this may also further disrupt the oscillations of the microbial rhythm. This further implies that the gut microbiota exhibits diurnal oscillations in both composition and function, with these fluctuations being regulated by the nutrient composition of the host’s diet.

## Effects of Microbiota on the Circadian Rhythms of Host

**FIGURE 3:**
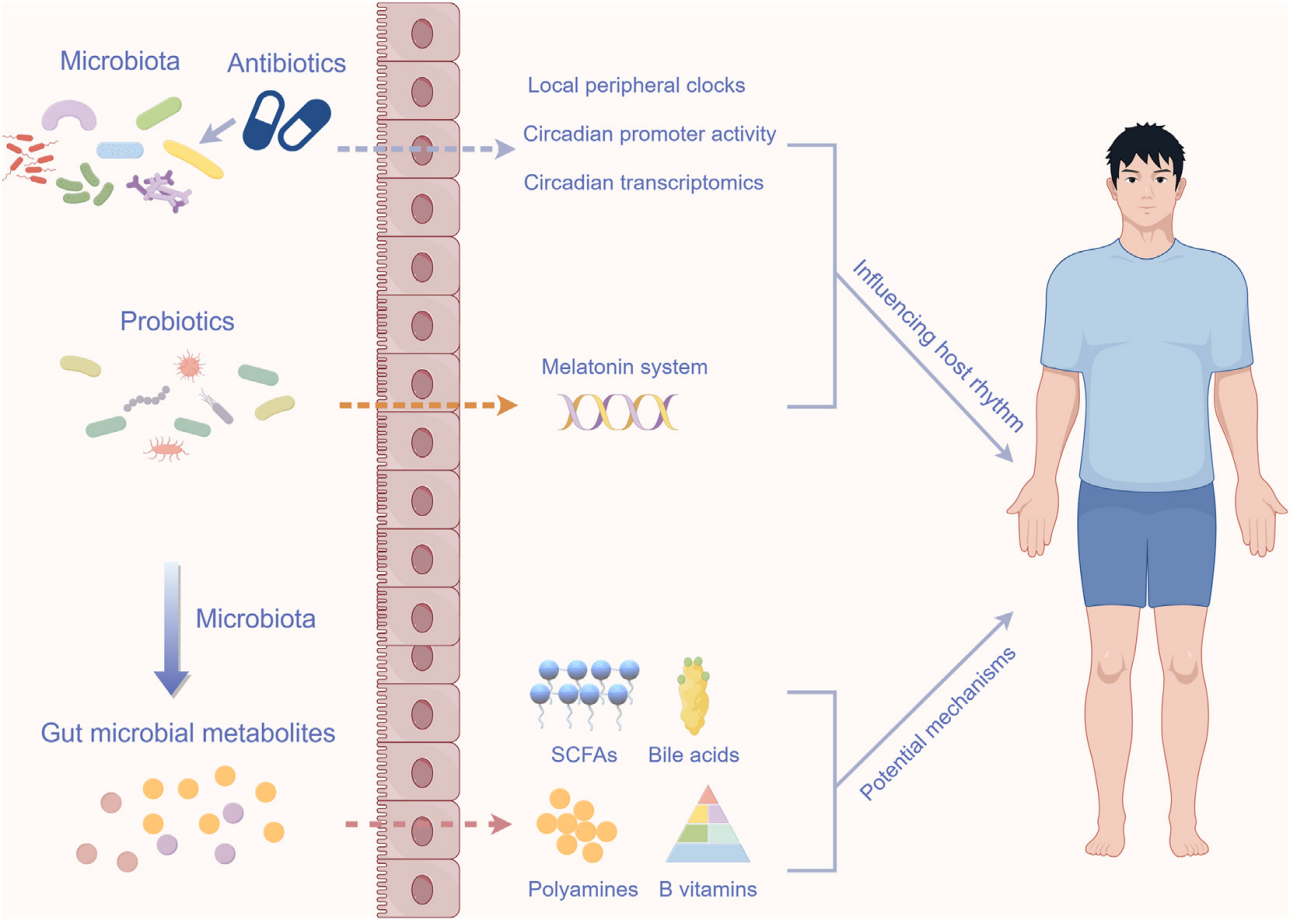
Gut microbiota influence host rhythms through various mechanisms. Both antibiotics and probiotic supplementation influence the rhythmic oscillations of the host. Additionally, gut microbiota further modulates the host rhythms through the production of various metabolites. This figure was created using Figdraw.com.

The gut microbiota is essential for immune, metabolic, and nervous system functions, making it crucial for overall health and well-being [22,60]. Growing evidence indicates that the gut microbiota plays a crucial role in regulating host metabolism, particularly by influencing and responding to host circadian rhythms [8,22,61,62] (Figure 3).

### Absence of the gut microbiota

A healthy gut microbiota (a balanced microbial ecosystem) ensures the host’s circadian rhythms, but its absence disrupts the host’s circadian gene expression [45,63]. Indeed, the administration of broad-spectrum antibiotics significantly reduces both the population of intestinal mucosal-associated bacteria and the oscillatory behavior [63]. Chromatin immunoprecipitation-sequencing analysis revealed discernible alters in the rhythmic promoter and enhancer activity in antibiotic-treated mice [63]. Furthermore, the oscillatory genes (i.e., *Il18*, *Reg3b*, and *Reg3g*) were altered in antibiotic-treated mice [63]. Similarly, the gut microbiota is also required for both the core clock of the liver and clock-controlled functions. Specifically, the expression patterns of *Bmal1*, *Per1/2*, and *Cry1* exhibit distinct profiles in germ-free mice [64]. Furthermore, the expression patterns of these output effectors (i.e., Dbp, Tef, and Bhlhb42) of the liver clock were disrupted in germ-free mice, leading to alterations in their gene expression rhythmic period [64]. Collectively, these findings suggest that gut microbiota influence the expression patterns of clock-related genes and output regulators.

Interestingly, the expression levels of core circadian clock genes in the mediobasal hypothalamus exhibited variations contingent upon the germ-free status and dietary conditions of the mice [17]. Furthermore, germ-free mice, irrespective of whether they are fed a low-fat or high-fat diet, demonstrate disrupted circadian clock gene expression in both central and hepatic systems in comparison with their conventionally raised counterparts [17]. This suggests that the absence of gut microbiota may alter the host’s circadian clock gene expression, potentially independent of dietary factors. Gut microbiota–mediated disruption of circadian rhythms generally leads to abnormal metabolic symptoms [45]. For example, antibiotic-induced depletion of the gut microbiota leads to hypercortisolism and insulin resistance [64]. Collectively, gut microbiota crucially regulates the host’s circadian rhythms.

### Probiotics

Probiotics may play an important role in improving the composition of gut microbiota to regulate host physiological functions [65]. Currently, the role of probiotics in regulating circadian rhythms has attracted much attention.

For example, *Bifidobacterium breve* strain CCFM1025 is reported to improve the body weight and food intake of the sleep deprivation mice [65]. Indeed, metabolites derived from *Bifidobacterium breve* may influence the striatal melatonin system, regulating the expression of circadian clock genes and thereby ameliorating circadian rhythm disruptions caused by sleep disorders [65]. Similarly, *Lactobacillus delbrueckii* was found to impact the bacterial composition at the genus level in the ileum, which might have implications for regulating gut function and host metabolism [66]. Furthermore, *Lactobacillus rhamnosus* may have additional effects on the gut microbiota. Although it may not significantly impact gut microbiota diversity within the first 24 h, it has been observed to enhance the abundances of melatonin receptor transcripts and proteins [67]. These findings from animal model studies suggest that probiotics’ regulation of circadian rhythms offers valuable insights for developing future treatments for circadian disorders in humans.

## Potential Mechanisms Through Which Microbiota Influence Host Circadian Rhythms

The gut microbiota, functioning as an active endocrine organ, generates various metabolites based on circadian rhythms and meal times [68]. These small molecule metabolites mediate signaling interactions within the host. There is also evidence that metabolic products from gut microbiota contribute to host circadian rhythms [2,22,61] (Figure 3). However, the precise mechanisms of their actions remain poorly understood and warrant further investigation.

### Short-chain fatty acids

SCFAs are well-established mechanisms in the interaction between gut microbiota and host metabolism. Germ-free mice are characterized by lowered SCFAs along with resistant to diet-induced obesity [22]. Supplementing the diet with acetate, propionate, butyrate, or the SCFAs admixture are directly reported to alleviate the weight gain [69]. The differential timing of SCFAs production and delivery have specific impacts on the host. Any alteration or disruption in the diurnal delivery pattern, potentially caused by circadian rhythm disturbances [22].

Diurnal oscillations of gut microbiota–derived SCFAs have been shown to influence circadian control of host metabolism. For instance, administering SCFAs and lactate orally significantly sped up the phase entrainment (the cycle or phase of the biorhythm is adjusted to align with periodic changes in the external environment) of PER2 rhythms in the kidney [70]. The addition of SCFAs like butyrate or acetate to hepatic organoids in vitro resulted in noteworthy alterations in PER2 and BMAL1 rhythms, manifesting as significant phase shifts alongside amplitude increases [17]. Similarly, oat fiber supplementation increases concentrations of SCFAs, reversing the disruption of the liver clock caused by a high-fat diet [71]. This further suggests that SCFAs are important contributors to the improvement of circadian rhythms in the host. Furthermore, gut microbiota–generated SCFAs, including acetate, isovaleric acid, propionate, and butyrate, entrain intestinal epithelial circadian rhythms through a histone deacetylase inhibition–dependent mechanism [72]. Therefore, SCFAs may act as synchronizers of circadian clocks and play a crucial role in the dynamic interplay between gut microbiota and host rhythms [17].

### Bile acids

Bile acids are key signaling molecules that regulate metabolism and connect circadian rhythms to the gut microbiome [[73], [74], [75], [76]]. For example, cholesterol 7α-hydroxylase (Cyp7α) and sterol 12α-hydroxylase (Cyp8b) are pivotal enzymes involved in the biosynthesis of bile acids within hepatic metabolic pathways, and the transcripts display circadian expression patterns in the liver [77]. Specifically, the expression profile of the Cyp7a transcript in wild-type mice exhibits circadian rhythms [77]. In contrast, in homozygous *Clock* mutant mice, the expression profile also demonstrates circadian rhythms, albeit with a reduced amplitude [77]. However, the expression profile of Cyp8b mRNA in homozygous *Clock* mutant mice becomes arrhythmic [77]. This indicates that the circadian regulation of Cyp7a and Cyp8b may involve the coordinated action of multiple clock transcription factors.

Furthermore, certain bile acids have been shown to inhibit the activation of the circadian transcription factor and PPARγ. Inhibition of this enzyme mediates the relationship between a high-fat diet and subsequent changes in the hepatic oscillators [58,78]. Similar studies have identified a role for bile salt hydrolase in the regulation of host circadian gene expression [79]. Unconjugated bile acids, generated by the bile salt hydrolase activity of the gut microbiota, have been proposed as potential regulators of circadian gene expression in the host [79]. Specifically, these unconjugated bile acids have been shown to enhance the expression levels of the *Clock* and *Arntl* genes, which are integral to circadian rhythms [79].

Furthermore, the oral administration of unconjugated bile acids modulates the expression of circadian clock genes in the ileum, colon, and liver of mice, thereby influencing hepatic circadian regulators such as Dbp, as well as associated genes including *Per2*, *Per3*, and *Cry2* [79]. Microbial metabolites, particularly unconjugated bile acids, can affect host metabolism and clock gene expression. Notably, conjugated and unconjugated bile acids follow different rhythms in human enterohepatic circulation [80]. Specifically, the transintestinal flux of conjugated bile acids postprandially elevates circulating concentrations of fibroblast growth factor 19, which subsequently inhibits bile acid synthesis [80]. Additionally, the late-night peaks of unconjugated bile acids indicate a nonpostprandial diurnal variation in human gut microbiota [80]. These studies indicate a potential circadian influence of particular bile acids on a complex within the host organism. Nonetheless, additional research is required to corroborate these findings.

### Other microbial metabolites

Apart from SCFAs and bile acids, other microbial metabolites may also impact host circadian rhythms. Notably, polyamines, as pleiotropic signaling molecules, exhibit diurnal fluctuations. In cultured cells and animal models, polyamines modulate the circadian rhythm by altering the interaction between Per2 and Cry1 [81]. Additionally, the dissimilatory sulfite reductase, predominantly found in deltaproteobacteria, produces hydrogen sulfide in the distal colon [82], which can delay the expression of Bmall in mouse liver organoids [17].

Certain B vitamins have been associated, either directly or indirectly, with circadian rhythms [83]. Although vitamins are typically obtained through dietary intake, genome annotations predict that 40%–65% of the 256 human gut microbes produce each of the 8 B vitamins [84]. For instance, *Bacteroides thetaiotaomicron* binds dietary corrinoids, which are precursors to vitamin B-12, and makes them available to the microbiota [84]. Collectively, host-regulated conditions—encompassing endogenous circadian control, diet, feeding times, and other factors—can induce alterations in microbial community structure and activity. Consequently, microbial metabolic activity plays a crucial role in regulating circadian rhythms, especially through the production of SCFAs.

## Disruption of Microbial and Host Circadian Rhythms: Health Implications

In mammals, including humans, lipid homeostasis is integral to the maintenance of metabolic health. Research findings indicate that functional circadian oscillators are essential for sustaining lipid homeostasis and, consequently, metabolic health [4]. However, disruptions or dysfunctions in circadian rhythms significantly impact host lipid metabolism and expedite the onset of metabolic disorders, including obesity, diabetes, and cardiovascular disease [4,7]. Notably, the gut microbiota possesses the capability to both convert and synthesize lipids, as well as to degrade dietary lipids, thereby producing secondary metabolites with host-modulating properties [85,86]. The role of lipid signaling in host–microbiome interactions has been relatively underexplored; however, recent findings indicate that interactions between microbiota and host lipid metabolism may significantly influence the regulation of the circadian clock [79,85]. Thus, given the influence of both microbiota–host lipid metabolism and circadian rhythms in host physiologic processes, it is crucial to study the interplay between these 2 systems in health and metabolic diseases.

### Obesity

Manipulating the gut microbiome can influence obesity by altering the host circadian clock [68]. To date, obesity research has identified the most significant association between circadian rhythms and the gut microbiota in the context of disease [2,17,27,[87], [88], [89]]. The expression of central and hepatic circadian clock genes is significantly impaired in germ-free mice fed a low or high-fat diet, and these mice do not gain weight [17]. Mice with the *Clock* mutation exhibit weaker daily eating patterns, impaired glucose tolerance, and reduced insulin secretion [90,91]. Furthermore, studies on *Clock*-mutant mice illustrate the impact of circadian clock disruption on the gut microbiota, which subsequently contributes to the development of obesity [28,90].

Recent research increasingly underscores the significant interplay between obesity, microbiota, and circadian rhythms, particularly emphasizing the influence of dietary patterns and composition on metabolism and gut microbiota [92]. For instance, a high-fat diet has been observed to induce lipid accumulation and disrupt gut microbiota homeostasis in murine models. In contrast, the administration of oral melatonin has been shown to mitigate lipid accumulation and improve gut microbiota composition [87]. Specifically, the administration of a high-fat diet in mice results in a reduction of SCFAs, whereas melatonin treatment enhances the synthesis of acetic acid [87]. Sleeve gastrectomy has been demonstrated to ameliorate metabolic disorders, an effect that is mediated by alterations in the gut microbiota [88]. Similarly, high-fat diets primarily drive microbial oscillators that disrupt host metabolic homeostasis, resulting in arrhythmic host *Reg3g* expression, which secondarily drives key gut microbial abundance and oscillation [5]. Accumulating evidence reveals that the gut microbiota significantly impacts the regulation of host peripheral clocks [58]. Intriguingly, complex interactions may exist between calorie intake, obesity, the microbiome, and circadian rhythms. For example, compared with individuals who consumed a high-calorie dinner, those who ate a high-calorie breakfast exhibited lower intake at dinner, reduced weight gain, less adipose tissue, and lower concentrations of fasting blood sugar, triglycerides, and insulin [93]. Furthermore, mice fed a high-fat diet during inactive periods (light) gained weight more rapidly than those fed high-fat diet only during active periods (dark) [94]. Therefore, it is imperative to devote additional scholarly attention to the role of circadian rhythms in the regulation of obesity.

### Diabetes

Emerging evidence suggests a significant association between gut microbiota and metabolic health, particularly in relation to alterations in the microbiota profile observed in individuals with diabetes [95]. For instance, research has demonstrated that patients with type 1 or type 2 diabetes are associated with alterations in gut microbiota composition when compared with healthy individuals, as evidenced by studies conducted in both humans and animal models [2]. Additionally, the untargeted metabolomics in blood plasma revealed changes in the diurnal patterns of metabolic pathways influenced by gut bacteria. It is suggested that diabetes disrupts the rhythm of gut microbiota oscillations, which may impact the temporal regulation of host metabolic pathways. Similarly, individuals with type 2 diabetes exhibited reduced amplitude oscillations in core clock genes (only 1.8% of expressed genes of 16,818) compared with healthy controls (8.4% of expressed genes of 1421) [89].

Notably, mice with KO mutations in the *Clock* and *Bmal1* genes demonstrate impaired glucose tolerance and diminished insulin secretion, thereby establishing a connection between circadian rhythm genes and the pathogenesis of diabetes. Additionally, these mice show defects in pancreatic islet size and proliferation that worsen with age [91]. Furthermore, arrhythmic gut microbiota signatures predict risk of type 2 diabetes [95]. Specifically, a study identified that certain members of the microbiota demonstrate 24-hour oscillations in their relative abundance. Consequently, this suggests a potential functional linkage between circadian rhythms and the microbiota in the context of diabetes.

### Cardiovascular disease

Recent research has revealed surprising interactions between gut microbiota rhythms and the host, altering risk of cardiovascular disease, a condition long linked to diet [96,97]. For instance, murine models of cardiovascular disease exhibit more rapid recovery when maintained under 24-h light–dark cycles, as opposed to conditions with disrupted circadian rhythms, which have been shown to exacerbate cardiac pathology [98]. Furthermore, mice harboring a point mutation in the circadian regulatory gene casein kinase-1ε demonstrated pronounced cardiomyopathy, extensive fibrosis, and renal pathology characterized by proteinuria, culminating in premature mortality [98]. However, the mice were ameliorated by reestablishing them in light–dark cycle, which was congruent with their genotype [98]. Similarly, short-term circadian misalignment, which involves inverting behavioral and environmental cycles for 3 days, has been shown to have adverse effects on cardiovascular disease risk factors in healthy adults [99].

Interestingly, dietary interventions involving high fiber intake and acetate supplementation have been shown to modulate gut microbiota composition and confer protective effects against hypertension and heart failure in murine models [100]. The beneficial impact of fiber and acetate may lead to an increased abundance of *Bacteroides acidifaciens*, promote circadian rhythm regulation through the upregulation of circadian genes and downregulate EGRL, a key cardiovascular regulator implicated in cardiorenal fibrosis and inflammation [100]. Furthermore, a metabolite derived from gut microbes, phenylacetylglutamine, has been shown to enhance the potential for thrombosis, which can lead to heart disease [101]. In summary, gut microbiota and circadian rhythms may mutually influence each other and modulate disease severity.

## Conclusion and Future Perspective

Over the past few decades, increasing evidence supports 2-way interaction between circadian rhythms and gut microbiota. However, the specific mechanisms involved remain unknown. The gut microbiota’s composition and function vary with the diurnal cycle, aligning with the host’s circadian rhythm. In turn, a complex microbiota and its metabolites are crucial for optimal regulation of the host’s circadian rhythm. However, disruption of the cyclical microbiota–host interactions leads to disorders of host metabolism and consequently to metabolic diseases [4,7]. Indeed, SCFAs and microbial-modified bile acids are potential mediators of interactions between gut microbial and circadian rhythms, affecting host metabolism and energy balance networks. However, how do these mediators accomplish signaling? Furthermore, the mechanism behind gut microbial rhythms is still unknown. Apart from light, diet, sex, and other known factors that affect host–microbial rhythms, what other factors may alter microbial rhythms? Extensive research has been conducted on diseases associated with the interplay between gut microbiota and circadian rhythms. However, the contribution of gut microbial rhythmicity to host well-being lacks clarity, and the regulatory impact of these influencers on specific microbial taxa dynamics requires further exploration. Therefore, more research is required to explore how microbiota and circadian rhythms influence host metabolism and interact in metabolic disorders. Future research will prominently focus on the interplay between gut microbiota, the circadian clock, and host metabolism.

## Author contributions

The authors’ responsibilities were as follows – MZ, WT, JY: conceived the study and wrote the original draft; XL, HL, QH, ZC: performed investigation and formal analysis; MZ, WT, JY: supervised the study; CZ: reviewed and edited the manuscript; and all authors: read and approved the final manuscript.

## Data availability

Data sharing is not applicable to this article as no new data were created or analyzed in this study.

## Funding

This work was supported by the Key Research and Development Program of Hunan Province (2023NK2018); the Scientific Research Fund of Hunan Provincial Education Department (22A0154); and “Huxiang Young Talents Plan” Project of Hunan Province (2022RC1157)

## Conflict of interest

JY reports financial support was provided by the Key Research and Development Program of Hunan Province, the Scientific Research Fund of Hunan Provincial Education Department, and Huxiang Young Talents Plan Project of Hunan Province. All other authors report no conflicts of interest.

## References

[1] Petrenko V., Sinturel F., Riezman H., Dibner C. (2023). Lipid metabolism around the body clocks. Prog. Lipid Res..

[2] Teichman E.M., O’Riordan K.J., Gahan C.G.M., Dinan T.G., Cryan J.F. (2020). When rhythms meet the blues: circadian interactions with the microbiota-gut-brain axis. Cell Metab..

[3] Frazier K., Manzoor S., Carroll K., DeLeon O., Miyoshi S., Miyoshi J. (2023). Gut microbes and the liver circadian clock partition glucose and lipid metabolism. J. Clin. Invest..

[4] Sinturel F., Spaleniak W., Dibner C. (2022). Circadian rhythm of lipid metabolism. Biochem. Soc. Trans..

[5] Frazier K., Kambal A., Zale E.A., Pierre J.F., Hubert N., Miyoshi S. (2022). High-fat diet disrupts REG3γ and gut microbial rhythms promoting metabolic dysfunction. Cell Host Microbe.

[6] Zhen Y., Ge L., Xu Q., Hu L., Wei W., Huang J. (2022). Normal light-dark and short-light cycles regulate intestinal inflammation, circulating short-chain fatty acids and gut microbiota in period2 gene knockout mice. Front. Immunol..

[7] Li Y., Ma J., Yao K., Su W., Tan B., Wu X. (2020). Circadian rhythms and obesity: timekeeping governs lipid metabolism. J. Pineal Res..

[8] Bishehsari F., Voigt R.M., Keshavarzian A. (2020). Circadian rhythms and the gut microbiota: from the metabolic syndrome to cancer. Nat. Rev. Endocrinol..

[9] Aggarwal N., Kitano S., Puah G.R.Y., Kittelmann S., Hwang I.Y., Chang M.W. (2023). Microbiome and human health: current understanding, engineering, and enabling technologies. Chem. Rev..

[10] Wang Y., He F., Liu B., Wu X., Han Z., Wang X. (2024). Interaction between intestinal mycobiota and microbiota shapes lung inflammation. Imeta.

[11] Xiao Y., Zou H., Li J., Song T., Lv W., Wang W. (2022). Impact of quorum sensing signaling molecules in gram-negative bacteria on host cells: current understanding and future perspectives. Gut Microbes.

[12] Zhou X., Liu Y., Xiong X., Chen J., Tang W., He L. (2022). Intestinal accumulation of microbiota-produced succinate caused by loss of microRNAs leads to diarrhea in weanling piglets. Gut Microbes.

[13] He L., Zhou X., Liu Y., Zhou L., Li F. (2022). Fecal miR-142a-3p from dextran sulfate sodium-challenge recovered mice prevents colitis by promoting the growth of *Lactobacillus reuteri*. Mol. Ther..

[14] Zhou X., He Y., Chen J., Xiong X., Yin J., Liang J. (2023). Colonic phosphocholine is correlated with *Candida tropicalis* and promotes diarrhea and pathogen clearance. NPJ Biofilms Microbiomes.

[15] Voigt R.M., Forsyth C.B., Green S.J., Engen P.A., Keshavarzian A. (2016). Circadian rhythm and the gut microbiome. Int. Rev. Neurobiol..

[16] Sharma S.A., Oladejo S.O., Kuang Z. (2025). Chemical interplay between gut microbiota and epigenetics: Implications in circadian biology. Cell Chem. Biol..

[17] Leone V., Gibbons S.M., Martinez K., Hutchison A.L., Huang E.Y., Cham C.M. (2015). Effects of diurnal variation of gut microbes and high-fat feeding on host circadian clock function and metabolism. Cell Host Microbe.

[18] Takahashi J.S. (2017). Transcriptional architecture of the mammalian circadian clock. Nat Rev. Genet..

[19] Lotti S., Dinu M., Colombini B., Amedei A., Sofi F. (2023). Circadian rhythms, gut microbiota, and diet: possible implications for health. Nutr. Metab. Cardiovasc. Dis..

[20] Speksnijder E.M., Bisschop P.H., Siegelaar S.E., Stenvers D.J., Kalsbeek A. (2024). Circadian desynchrony and glucose metabolism. J. Pineal Res..

[21] Patke A., Young M.W., Axelrod S. (2020). Molecular mechanisms and physiological importance of circadian rhythms. Nat. Rev. Mol. Cell Biol..

[22] Frazier K., Chang E.B. (2020). Intersection of the gut microbiome and circadian rhythms in metabolism, Trends Endocrinol. Metab..

[23] Wang H., Zhang H., Su Y. (2022). New insights into the diurnal rhythmicity of gut microbiota and its crosstalk with host circadian rhythm. Animals.

[24] Xie X., Xu H., Shu R., Sun L., Zhang M., Hu Q. (2023). Clock gene Per3 deficiency disrupts circadian alterations of gut microbiota in mice. Acta Biochim. Biophys. Sin (Shanghai)..

[25] Liang X., Bushman F.D., FitzGerald G.A. (2015). Rhythmicity of the intestinal microbiota is regulated by gender and the host circadian clock. Proc. Natl. Acad. Sci. U.S.A..

[26] Segers A., Desmet L., Thijs T., Verbeke K., Tack J., Depoortere I. (2019). The circadian clock regulates the diurnal levels of microbial short-chain fatty acids and their rhythmic effects on colon contractility in mice. Acta Physiol. (Oxf)..

[27] Thaiss C.A., Zeevi D., Levy M., Zilberman-Schapira G., Suez J., Tengeler A.C. (2014). Transkingdom control of microbiota diurnal oscillations promotes metabolic homeostasis. Cell..

[28] Voigt R.M., Summa K.C., Forsyth C.B., Green S.J., Engen P., Naqib A. (2016). The circadian clock mutation promotes intestinal dysbiosis. Alcohol. Clin. Exp. Res..

[29] O’Neil D.S., Stewart C.J., Chu D.M., Goodspeed D.M., Gonzalez-Rodriguez P.J., Shope C.D. (2017). Conditional postnatal deletion of the neonatal murine hepatic circadian gene, Npas2, alters the gut microbiome following restricted feeding. Am. J. Obstet. Gynecol..

[30] Valeri F., Endres K. (2021). How biological sex of the host shapes its gut microbiota. Front. Neuroendocrinol..

[31] Org E., Mehrabian M., Parks B.W., Shipkova P., Liu X., Drake T.A. (2016). Sex differences and hormonal effects on gut microbiota composition in mice. Gut Microbes.

[32] Jiang Y., Li S., Xu W., Ying J., Qu Y., Jiang X. (2022). Critical roles of the circadian transcription factor BMAL1 in reproductive endocrinology and fertility. Front. Endocrinol..

[33] Kim H.-J., Moon C.M., Kang J.L., Park E.-M. (2021). Aging effects on the diurnal patterns of gut microbial composition in male and female mice. Korean J. Physiol. Pharmacol..

[34] Yin J., Li Y., Han H., Ma J., Liu G., Wu X. (2020). Administration of exogenous melatonin improves the diurnal rhythms of the gut microbiota in mice fed a high-fat diet. mSystems.

[35] Ye Y., Xu H., Xie Z., Wang L., Sun Y., Yang H. (2020). Time-restricted feeding reduces the detrimental effects of a high-fat diet, possibly by modulating the circadian rhythm of hepatic lipid metabolism and gut microbiota. Front. Nutr..

[36] Pearson J.A., Wong F.S., Wen L. (2020). Crosstalk between circadian rhythms and the microbiota. Immunology.

[37] Fan L., Xia Y., Wang Y., Han D., Liu Y., Li J. (2023). Gut microbiota bridges dietary nutrients and host immunity. Sci. China Life Sci..

[38] Wu G., Tang W., He Y., Hu J., Gong S., He Z. (2018). Light exposure influences the diurnal oscillation of gut microbiota in mice. Biochem. Biophys. Res. Commun..

[39] Hong F., Pan S., Xu P., Xue T., Wang J., Guo Y. (2020). Melatonin orchestrates lipid homeostasis through the hepatointestinal circadian clock and microbiota during constant light exposure. Cells.

[40] Brooks J.F., Behrendt C.L., Ruhn K.A., Lee S., Raj P., Takahashi J.S. (2021). The microbiota coordinates diurnal rhythms in innate immunity with the circadian clock. Cell.

[41] Heddes M., Altaha B., Niu Y., Reitmeier S., Kleigrewe K., Haller D. (2022). The intestinal clock drives the microbiome to maintain gastrointestinal homeostasis. Nat. Commun..

[42] Deaver J.A., Eum S.Y., Toborek M. (2018). Circadian disruption changes gut microbiome taxa and functional gene composition. Front. Microbiol..

[43] Daas M.C., Roos N.M.D. (2021). Intermittent fasting contributes to aligned circadian rhythms through interactions with the gut microbiome. Benef. Microbes..

[44] Zeb F., Wu X., Feng Q. (2021). Effect of time restricted feeding on metabolic risk and circadian rhythm associated with gut microbiome in healthy males. Curr. Dev. Nutr..

[45] Choi H., Rao M.C., Chang E.B. (2021). Gut microbiota as a transducer of dietary cues to regulate host circadian rhythms and metabolism. Nat. Rev. Gastroenterol. Hepatol..

[46] Zarrinpar A., Chaix A., Yooseph S., Panda S. (2014). Diet and feeding pattern affect the diurnal dynamics of the gut microbiome. Cell Metab..

[47] Zeb F., Wu X., Chen L., Fatima S., Li M. (2020). Effect of time restricted feeding on metabolic risk and circadian rhythm associated with gut microbiome in healthy males. Br. J. Nutr..

[48] Nakahata Y., Kaluzova M., Grimaldi B., Sahar S., Hirayama J., Chen D. (2008). The NAD+-dependent deacetylase SIRT1 modulates CLOCK-mediated chromatin remodeling and circadian control. Cell.

[49] Sato S., Solanas G., Peixoto F.O., Bee L., Symeonidi A., Schmidt M.S. (2017). Circadian reprogramming in the liver identifies metabolic pathways of aging. Cell.

[50] Asher G., Sassone-Corsi P. (2015). Time for food: the intimate interplay between nutrition, metabolism, and the circadian clock. Cell..

[51] Yan R., Yang C.S., Zhang X. (2021). Maintain host health with time-restricted eating and phytochemicals: a review based on gut microbiome and circadian rhythm. Trends Food Sci. Technol..

[52] Cui Y., Li S., Yin Y., Li X., Li X. (2022). Daytime restricted feeding promotes circadian desynchrony and metabolic disruption with changes in bile acids profiles and gut microbiota in C57BL/6 male mice. J. Nutr. Biochem..

[53] Lewis P., Oster H., Korf H.W., Foster R.G., Erren T.C. (2020). Food as a circadian time cue—evidence from human studies. Nat. Rev. Endocrinol..

[54] Gutierrez Lopez D.E., Lashinger L.M., Weinstock G.M., Bray M.S. (2021). Circadian rhythms and the gut microbiome synchronize the host's metabolic response to diet. Cell Metab..

[55] la Fleur S.E., Blancas-Velazquez A.S., Stenvers D.J., Kalsbeek A. (2024). Circadian influences on feeding behavior. Neuropharmacology.

[56] Moreira Gobis M.d.L., Goulart de Souza-Silva T., de Almeida Paula H.A. (2024). The impact of a western diet on gut microbiota and circadian rhythm: a comprehensive systematic review of in vivo preclinical evidence. Life Sci..

[57] Wollmuth E.M., Angert E.R. (2023). Microbial circadian clocks: host-microbe interplay in diel cycles. BMC Microbiol.

[58] Murakami M., Tognini P., Liu Y., Eckel-Mahan K.L., Baldi P., Sassone-Corsi P. (2016). Gut microbiota directs PPARγ-driven reprogramming of the liver circadian clock by nutritional challenge. EMBO Rep.

[59] Xin H., Zhang J., Huang R., Li L., Lam S.M., Shui G. (2022). Circadian signatures of adipose tissue in diet-induced obesity. Front. Physiol..

[60] Zhang Y., Li Y., Barber A.F., Noya S.B., Williams J.A., Li F. (2023). The microbiome stabilizes circadian rhythms in the gut. Proc. Natl. Acad. Sci. U. S. A..

[61] Schmalle V., Lorentz A. (2020). Role of the microbiota in circadian rhythms of the host. Chronobiol. Int..

[62] Murakami M., Tognini P. (2019). The circadian clock as an essential molecular link between host physiology and microorganisms. Front. Cell. Infect. Microbiol..

[63] Thaiss C.A., Levy M., Korem T., Dohnalová L., Shapiro H., Jaitin D.A. (2016). Microbiota diurnal rhythmicity programs host transcriptome oscillations. Cell..

[64] Montagner A., Korecka A., Polizzi A., Lippi Y., Blum Y., Canlet C. (2016). Hepatic circadian clock oscillators and nuclear receptors integrate microbiome-derived signals. Sci. Rep..

[65] Tian P., Hou Y., Wang Z., Jiang J., Qian X., Qu Z. (2024). Probiotics administration alleviates cognitive impairment and circadian rhythm disturbance induced by sleep deprivation. Food Sci. Hum. Wellness.

[66] Luo W., Yin Z., Zhang M., Huang X., Yin J. (2024). Dietary *Lactobacillus delbrueckii* affects ileal bacterial composition and circadian rhythms in pigs. Animals (Basel).

[67] Lutfi E., Basili D., Falcinelli S., Morillas L., Carnevali O., Capilla E. (2021). The probiotic *Lactobacillus rhamnosus* mimics the dark-driven regulation of appetite markers and melatonin receptors’ expression in zebrafish (*Danio rerio*) larvae: understanding the role of the gut microbiome. Comp. Biochem. Physiol. B Biochem. Mol. Biol..

[68] Schugar R.C., Gliniak C.M., Osborn L.J., Massey W., Sangwan N., Horak A. (2022). Gut microbe-targeted choline trimethylamine lyase inhibition improves obesity via rewiring of host circadian rhythms. Elife.

[69] Lu Y., Fan C., Li P., Lu Y., Chang X., Qi K. (2016). Short chain fatty acids prevent high-fat-diet-induced obesity in mice by regulating G protein-coupled receptors and gut microbiota. Sci. Rep..

[70] Tahara Y., Yamazaki M., Sukigara H., Motohashi H., Sasaki H., Miyakawa H. (2018). Gut microbiota-derived short chain fatty acids induce circadian clock entrainment in mouse peripheral tissue. Sci. Rep..

[71] Han S., Gao H., Song R., Zhang W., Li Y., Zhang J. (2021). Oat fiber modulates hepatic circadian clock via promoting gut microbiota-derived short chain fatty acids. J. Agric. Food Chem..

[72] Fawad J.A., Luzader D.H., Hanson G.F., Moutinho T.J., Mckinney C.A., Mitchell P.G. (2022). Histone deacetylase inhibition by gut microbe-generated short-chain fatty acids entrains intestinal epithelial circadian rhythms. Gastroenterology.

[73] Duparc T., Plovier H., Marrachelli V.G., Van Hul M., Essaghir A., Ståhlman M. (2017). Hepatocyte MyD88 affects bile acids, gut microbiota and metabolome contributing to regulate glucose and lipid metabolism. Gut.

[74] Yu Z., Yang J., Xiang D., Li G., Liu D., Zhang C. (2020). Circadian rhythms and bile acid homeostasis: a comprehensive review. Chronobiol. Int..

[75] Yang Y., Zhang J. (2020). Bile acid metabolism and circadian rhythms. Am. J. Physiol. Gastrointest. Liver Physiol..

[76] Jia W., Li Y., Cheung K.C.P., Zheng X. (2024). Bile acid signaling in the regulation of whole body metabolic and immunological homeostasis. Sci. China Life Sci..

[77] Noshiro M., Usui E., Kawamoto T., Kubo H., Fujimoto K., Furukawa M. (2007). Multiple mechanisms regulate circadian expression of the gene for cholesterol 7α-hydroxylase (Cyp7a), a key enzyme in hepatic bile acid biosynthesis. J. Biol. Rhythms..

[78] Oh H.Y.P., Visvalingam V., Wahli W. (2019). The PPAR–microbiota–metabolic organ trilogy to fine-tune physiology. FASEB J..

[79] Govindarajan K., MacSharry J., Casey P.G., Shanahan F., Joyce S.A., Gahan C.G.M. (2016). Unconjugated bile acids influence expression of circadian genes: a potential mechanism for microbe-host crosstalk. PLoS One.

[80] Al-Khaifi A., Straniero S., Voronova V., Chernikova D., Sokolov V., Kumar C. (2018). Asynchronous rhythms of circulating conjugated and unconjugated bile acids in the modulation of human metabolism. J. Intern. Med..

[81] Zwighaft Z., Aviram R., Shalev M., Rousso-Noori L., Kraut-Cohen J., Golik M. (2015). Circadian clock control by polyamine levels through a mechanism that declines with age. Cell Metab..

[82] Carbonero F., Benefiel A.C., Alizadeh-Ghamsari A.H., Gaskins H.R. (2012). Microbial pathways in colonic sulfur metabolism and links with health and disease. Front. Physiol..

[83] Wolf G. (2002). Three vitamins are involved in regulation of the circadian rhythm. Nutr. Rev..

[84] Magnúsdóttir S., Ravcheev D., de Crécy-Lagard V., Thiele I. (2015). Systematic genome assessment of B-vitamin biosynthesis suggests co-operation among gut microbes. Front. Genet..

[85] Brown E.M., Clardy J., Xavier R.J. (2023). Gut microbiome lipid metabolism and its impact on host physiology. Cell Host Microbe.

[86] Ma L., Lyu W., Song Y., Chen K., Lv L., Yang H. (2023). Anti-inflammatory effect of *Clostridium butyricum*-derived extracellular vesicles in ulcerative colitis: impact on host microRNAs expressions and gut microbiome profiles. Mol. Nutr. Food Res..

[87] Yin J., Li Y., Han H., Chen S., Gao J., Liu G. (2018). Melatonin reprogramming of gut microbiota improves lipid dysmetabolism in high-fat diet-fed mice. J. Pineal Res..

[88] Shao Y., Shen Q., Hua R., Evers S.S., He K., Yao Q. (2018). Effects of sleeve gastrectomy on the composition and diurnal oscillation of gut microbiota related to the metabolic improvements. Surg. Obes. Relat. Dis..

[89] Stenvers D.J., Jongejan A., Atiqi S., Vreijling J.P., Limonard E.J., Endert E. (2019). Diurnal rhythms in the white adipose tissue transcriptome are disturbed in obese individuals with type 2 diabetes compared with lean control individuals. Diabetologia.

[90] Turek F.W., Joshu C., Kohsaka A., Lin E., Ivanova G., McDearmon E. (2005). Obesity and metabolic syndrome in circadian Clock mutant mice. Science.

[91] Marcheva B., Ramsey K.M., Buhr E.D., Kobayashi Y., Su H., Ko C.H. (2010). Disruption of the clock components CLOCK and BMAL1 leads to hypoinsulinaemia and diabetes. Nature.

[92] Li G., Xie C., Lu S., Nichols R.G., Tian Y., Li L. (2017). Intermittent fasting promotes white adipose browning and decreases obesity by shaping the gut microbiota. Cell Metab..

[93] Jakubowicz D., Barnea M., Wainstein J., Froy O. (2013). High caloric intake at breakfast vs. dinner differentially influences weight loss of overweight and obese women. Obesity (Silver Spring).

[94] Hepler C., Weidemann B.J., Waldeck N.J., Marcheva B., Cedernaes J., Thorne A.K. (2022). Time-restricted feeding mitigates obesity through adipocyte thermogenesis. Science..

[95] Reitmeier S., Kiessling S., Clavel T., List M., Almeida E.L., Ghosh T.S. (2020). Arrhythmic gut microbiome signatures predict risk of type 2 diabetes. Cell Host Microbe.

[96] Brown J.M., Hazen S.L. (2018). Microbial modulation of cardiovascular disease. Nat. Rev. Microbiol..

[97] Jie Z., Xia H., Zhong S.-L., Feng Q., Li S., Liang S. (2017). The gut microbiome in atherosclerotic cardiovascular disease. Nat. Commun..

[98] Martino T.A., Tata N., Belsham D.D., Chalmers J., Straume M., Lee P. (2007). Disturbed diurnal rhythm alters gene expression and exacerbates cardiovascular disease with rescue by resynchronization. Hypertension.

[99] Morris C.J., Purvis T.E., Hu K., Scheer F.A.J.L. (2016). Circadian misalignment increases cardiovascular disease risk factors in humans. Proc. Natl. Acad. Sci. U. S. A..

[100] Marques F.Z., Nelson E., Chu P.-Y., Horlock D., Fiedler A., Ziemann M. (2017). High-fiber diet and acetate supplementation change the gut microbiota and prevent the development of hypertension and heart failure in hypertensive mice. Circulation.

[101] Nemet I., Saha P.P., Gupta N., Zhu W., Romano K.A., Skye S.M. (2020). A cardiovascular disease-linked gut microbial metabolite acts via adrenergic receptors. Cell.

